# Non-Alcoholic Fatty Liver Disease Is not Related to the Incidence of Diabetic Nephropathy in Type 2 Diabetes

**DOI:** 10.3390/ijms131114698

**Published:** 2012-11-12

**Authors:** Yu-Tao Zhan, Chuan Zhang, Li Li, Chun-Shan Bi, Xin Song, Shu-Tian Zhang

**Affiliations:** 1 Department of Gastroenterology, Beijing Tongren Hospital, Capital Medical University, Beijing 100730, China; E-Mails: yutaozhan@263.net (Y.-T.Z.); doctorlili008@163.com (L.L.); bcszhr@163.com (C.-S.B.); 2 Department of Gastroenterology, Beijing Chaoyang Hospital Jingxi Campus, Capital Medical University, Beijing 100430, China; E-Mail: zhangchuan71@hotmail.com; 3 Department of Clinic Laboratory, Beijing Tongren Hospital, Capital Medical University, Beijing 100730, China; E-Mail: songxin_1103@163.com; 4 Department of Gastroenterology, Beijing Friendship Hospital, Capital Medical University, Beijing 100050, China

**Keywords:** non-alcoholic fatty liver disease, diabetic nephropathy, diabetes

## Abstract

To analyze the association between non-alcoholic fatty liver disease (NAFLD) and the incidence of diabetic nephropathy in patients with type 2 diabetes, the incidence of diabetic nephropathy was assessed in 413 type 2 diabetic patients, by testing the 24 h urinary albumin excretion rate (UAER). The NAFLD was diagnosed based on patient’s medical history and liver ultrasound. The difference in diabetic nephropathy incidence between patients with and without NAFLD was tested by χ^2^. Multivariate logistic regression analysis was used to assess the factors associated with diabetic nephropathy among type 2 diabetic patients. Total 363 out of 413 type 2 diabetic patients were enrolled in this study. The incidences of NAFLD and diabetic nephropathy in participants were approximately 56% (202/363) and 38% (137/363) respectively, and there was no significant difference in the prevalence of diabetic nephropathy between patients with and without NAFLD (37.1% *vs.* 38.5%, *p* = 0.787). The duration of diabetes (odds ratio [OR] 1.065, 95% confidence interval [CI] 1.014–1.120, *p* = 0.012), waist circumference (OR 1.077, 95% CI 1.040–1.116, *p* = 0.000), and fasting blood glucose (FBG; OR 1.136, 95% CI 1.023–1.1262, *p* = 0.017) were significantly associated with diabetic nephropathy, whereas sex, high blood pressure, total cholesterol (TC), triglyceride (TG), and ankle brachial pressure index (ABI) were not significantly associated with the disorder. The present results suggest that NAFLD is not related to the incidence of diabetic nephropathy in type 2 diabetes, but the duration of diabetes, waist circumference, and FBG are important factors for diabetic nephropathy in type 2 diabetes.

## 1. Introduction

NAFLD is the most common liver disease in the United States and other developed countries [1,2]. In the United States, about 30% of adults have NAFLD [3]. In the past, NAFLD was considered to be a benign liver disease. However, a recent growing body of evidence suggests that some patients with NAFLD may progress to nonalcoholic steatohepatitis (NASH), then cirrhosis and eventually hepatocellular carcinoma [4]. Moreover, NAFLD can promote the development and progression of diseases in other organs, which has attracted great interest. Several studies confirmed that NAFLD is associated with a higher prevalence of cardiovascular disease and it is an independent risk factor for cardiovascular disease in type 2 diabetes [5–7]. NAFLD also significantly increases the risk of diabetes [8,9]. Although recent studies on the association between NAFLD and chronic kidney disease (CKD) showed different results [10], whether or not NAFLD is an independent risk factor for diabetic nephropathy in type 2 diabetes patients in China remains unclear. UAER is the best available marker for the diagnosis of diabetic nephropathy [11,12]. Therefore, by testing UAER, the present study aims to reveal the association between NAFLD and diabetic nephropathy in type 2 diabetes patients in China.

## 2. Results and Discussion

### 2.1. Study Participants

**Figure 1:**
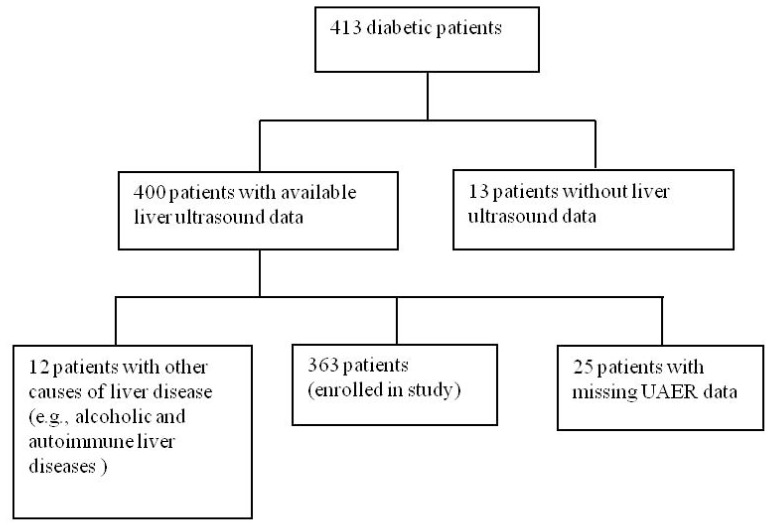
Diagram of the study design.

As shown in Figure 1, 413 type 2 diabetic patients were initially screened. Among them, excluded patients consisted of 13 subjects who did not have a liver ultrasound examination, 11 subjects (male) who consumed more than 20 g of alcohol per day, 1 subject who had autoimmune liver disease, and 25 subjects who did not have a UAER test. As a result, the remaining 363 patients met the inclusion criteria for further diagnosis of NAFLD, *i.e.*, hepatic steatosis without excessive alcohol consumption or other causes of chronic liver disease.

### 2.2. The Clinical Features and Biochemical Characteristics of Participants

The prevalence of NAFLD in present study was approximately 56% (202/363). Table 1 shows the clinical features and biochemical characteristics of the study group. Patients with NAFLD had higher BMI (*p* < 0.001), higher waist circumference (*p* < 0.001), higher TG (*p* < 0.001), lower high density lipoprotein (HDL, *p* < 0.001), and higher liver enzymes (*p* < 0.001) than patients without NAFLD. The sex, age, duration of diabetes, high blood pressure, FBG, HbA1C, TC, LDL cholesterol, and ABI did not differ between the groups.

### 2.3. Incidence of Diabetic Nephropathy in Participants

The incidence of diabetic nephropathy among the participants was about 38% (137/363), and the incidences of microalbuminuria and macroalbuminuria were approximately 25% (92/363) and 13% (47/363), respectively. As shown in Table 2, the incidences of diabetic nephropathy between patients with NAFLD and without NAFLD were not significantly different (*p >* 0.05). Neither were the incidences of microalbuminuria nor macroalbuminuria significantly different (*p >* 0.05) between patients with NAFLD and without NAFLD. These results suggest that NAFLD is not a risk factor for diabetic nephropathy in type 2 diabetes.

### 2.4. Risk Factor for Diabetic Nephropathy

As shown in Table 3, in multivariate regression analysis, the duration of diabetes (OR 1.065, 95% CI 1.014–1.120), waist circumference (OR 1.077, 95% CI 1.040–1.116), and FBG (OR 1.136, 95% CI 1.023–1.1262) was significantly associated with diabetic nephropathy, whereas sex, high blood pressure, TG, TC, and ABI were not significantly associated with the disorder.

## 3. Discussion

Type 2 diabetes is a common metabolic disease with a rising global prevalence. Diabetic nephropathy is a major complication of diabetes mellitus [13], and about 40% of patients with type 2 diabetes progress to diabetic nephropathy, the most common cause of chronic kidney failure and end-stage kidney disease in the developed world. With the increase of incidence of diabetes, such trend will continue [14]. Identifying the risk factors for diabetic nephropathy is important for the prevention and treatment of the disease. Type 2 diabetes is frequently accompanied by NAFLD. Approximately 70%–75% of type 2 diabetic patients have NAFLD [7,15]. As a result, it is of significance to know whether or not NAFLD is a risk factor for diabetic nephropathy in type 2 diabetes patients.

Recently, several studies have examined the prevalence of CKD in patients with NAFLD. The majority found that NAFLD is associated with increased risk of prevalent chronic kidney disease. The data has also shown that CKD and NAFLD share many important cardio-metabolic risk factors. However, that of the NAFLD is associated with CKD for sharing these risk factors or whether NAFLD itself contributes to the development of CKD independently without involving these factors remains unclear. In a large cohort study of type 2 diabetic individuals, Targher *et al*. found that patients with ultrasound-diagnosed NAFLD were associated with an increased prevalence of CKD, and such an association appeared to be independent of a broad spectrum of baseline confounding variables, such as traditional cardiovascular risk factors, diabetes duration, glycemic control, metabolic syndrome components, and uses of medications such as hypoglycemic, lipid-lowering, anti-hypertensive and anti-platelet drugs [16]. Hwang *et al*. also found that patients with ultrasound-diagnosed NAFLD had a greater prevalence of microalbuminuria compared with those patients with pre-diabetes or newly diagnosed diabetes but without NAFLD [17]. Multivariate logistic regression analysis revealed that NAFLD was associated with the presence of microalbuminuria independent from several potential confounders. These results suggest that NAFLD is an independent risk factor for CKD in type 2 diabetic patients; however, the intertwined mechanisms that link NAFLD and CKD remain unclear. Several pro-inflammatory/pro-coagulant mediators from the steatotic/inflamed liver or the insulin resistance induced by NAFLD itself and atherogenic dyslipidemia may have contributed to the pathogenesis of CKD-related NAFLD. However, in a small study that used liver biopsy to diagnose NAFLD, Manco *et al*. [18] failed to detect any significant differences in UAER between overweight/obese children with NAFLD and age- and sex-matched control children without NAFLD. To our knowledge, this study for the first time analyzed the association between NAFLD and diabetic nephropathy in Chinese patients with type 2 diabetes. The present study did not find any significant differences in the incidence of diabetic nephropathy between type 2 diabetic patients with NAFLD and without NAFLD and suggested that NAFLD is not a risk factor for diabetic nephropathy in Chinese type 2 diabetic patients. NAFLD includes simple fatty liver and NASH, a progressive form of NAFLD, where accumulation of excessive fat coexists with liver cell injury and inflammation. Only liver histopathological test, not ultrasound scanning, can distinguish NASH from simple fatty liver. Since the patents in this study were not examined by histopathological test, the relationship between NASH and CKD cannot be excluded and needs further studies. The contradictory results between ours and other investigators’ may be due to the racial difference between participants. The present study found that the duration of diabetes, waist circumference, and FBG were significantly associated with diabetic nephropathy. Thus, early diagnosis of diabetes, maintaining a normal waist circumference, and keeping blood sugar level as close to normal as possible are important in the prevention and treatment of diabetic nephropathy.

The present study has limitations. First, it was a cross-sectional retrospective study in which some indices or factors affecting albuminuria were missing or imperfect. While UAER is the best available marker for the diagnosis of diabetic nephropathy, glomerular filtration rate is another available diagnostic marker of diabetic nephropathy. Because the urine creatinine was not tested with the patients in this study, a glomerular filtration rate was not calculated; therefore, the relationship between glomerular filtration rate and NAFLD was not analyzed in this study. Second, the diagnosis of NAFLD was based on ultrasonography, a method that allows the detection of liver steatosis only when fat in liver exceeds 33%. The proton magnetic resonance spectroscopy (^1^H-MRS) is a precise evaluation method for liver steatosis; it is able to detect steatosis when liver fat exceeds 5.6%. However, ^1^H-MRS has some disadvantages, such as easily affected by breathing artifacts, complex post-imaging data analysis, and high cost. Therefore, ^1^H-MRS has not been widely applied clinically. As a result, the detection rate of NAFLD based on ultrasonography in this study may be lower than the actual incidence.

Although the present study is retrospective, many results are consistent with those reported previously. For example, the incidence rate of diabetic nephropathy in type 2 diabetic patients in this study (38%) is in agreement with that reported by Kumar *et al*. (~40%) [14]. Zachary *et al*. also reported that increased UAER was associated with greater waist circumference [19]. These results suggest that our results are valid and reliable.

## 4. Materials and Methods

### 4.1. Participants

This study carried out a retrospective observational analysis with a total of 413 type 2 diabetic patients in the Department of Endocrinology, Beijing Tongren Hospital, Capital Medical University, Beijing, China.

### 4.2. Clinical and Laboratory Measurements

#### 4.2.1. Human Body Indices

Human body indices included sex, age, duration of diabetes, height, body weight, waist circumference, BMI and systolic and diastolic blood pressures. The waist circumference was measured at the level of the umbilicus. The BMI was calculated by dividing body weight (kilograms) by height (meters) squared. The blood pressure was assessed with a standard mercury manometer.

#### 4.2.2. Biochemical Indices

Venous blood for biochemical indices was drawn from hospitalized patients in the morning after an over-night fast. Blood biochemical measurements were determined by standard laboratory procedures. The biochemical indices collected were as follows: FBG, glycosylated hemoglobin Alc (HbAlc), TC, TG, HDL, low density lipoprotein (LDL), alanine aminotransferase (ALT), aspartate aminotransferase (AST), and γ-glutamyltransferase (γ-GT). The abnormal ALT, AST, and γ-GT were defined as ALT > 40 U/L, AST > 40 U/L, and γ-GT > 50 U/L respectively.

### 4.3. Assessment of Diabetic Nephropathy

Twenty-four hour urine samples were collected for analyses of UAER. The patients with UAER < 20 μg/min were considered normoalbuminuric; the patients with UAER between 20 and 200 μg/min were classified as microalbuminuric; and the patients with UAER > 200 μg/min were considered to have macroalbuminuria. The patients with microalbuminuria or macroalbuminuria were considered to have DN [20].

### 4.4. Calculation of ABI

The ABI was calculated by dividing the systolic blood pressure at the ankle by the systolic blood pressures in the arm.

### 4.5. Diagnosis of NAFLD

Fatty liver was diagnosed by hepatic ultrasound scanning, which, as a part of the regular clinical assessment, was performed by a trained operator who was blind to all clinical and laboratory characteristics of patients. The diagnosis of hepatic steatosis was made in the presence of two or three abnormal findings on abdominal ultrasonography: diffusely increased echogenicity (“bright”) liver with liver echogenicity greater than kidney, with vascular blurring and deep attenuation of ultrasound signal [21]. NAFLD was diagnosed by referencing the guidelines for diagnosis and treatment of NAFLD published by the fatty liver and alcoholic liver disease study group of the Chinese liver disease association (2006) [22], *i.e.*, fatty liver patients without excessive alcohol consumption or other causes of chronic liver disease. The following exclusion criteria of NAFLD were applied: (1) patients with a history of drinking alcohol, or alcohol intake per day is more than 20 g in men and 10 g in women. Alcohol intake is calculated according to the following formula: the amount of alcohol (g) = alcohol consumption (mL) × alcohol concentration (%) × 0.8 (alcohol density); (2) patients were positive for hepatitis B or C viruses; (3) patients took liver disease-inducing drugs, such as tamoxifen, ethylamine iodine furosemide ketone, sodium valproate, methotrexate, and glucocorticoids; and (4) patients were positive for antinuclear or smooth muscle antibodies.

### 4.6. Statistical Analysis

The measurement data were presented as the means ± SD, and the enumeration data were expressed as percentages. The SPSS statistical package 11.5 was used for all statistical analyses. Values at *p* < 0.05 were considered to be statistically significant. Two groups of participants were compared by the unpaired *t*-test or Mann-Whitney test for measurement data (*t-*test for normally distributed variables, Mann-Whitney test for not normally distributed variables) and the χ^2^-test for count data. Multivariate logistic regression analysis was used to assess the factors associated with diabetic nephropathy among type 2 diabetic patients, and OR and 95% CI were calculated. In the logistic regression models, the duration of diabetes, waist circumference, FBG, sex, high blood pressure, TG, TC, and ABI were included as covariates.

## 5. Conclusions

To our knowledge, this is the first report on the association between NAFLD and diabetic nephropathy in type 2 diabetes patients in China. Our results have shown that NAFLD is not associated with the incidence of diabetic nephropathy in type 2 diabetes. The duration of diabetes, waist circumference, and FBG are important factors for diabetic nephropathy in Chinese type 2 diabetic patients. These results suggest that treatment of NAFLD is not important for preventing diabetic nephropathy, but early diagnosis and treatment of diabetes as well as reducing body weight may be beneficial in the prevention of diabetic nephropathy.

## Figures and Tables

**Table 1:** Clinical features and biochemical characteristics of participants.

Variables	With NAFLD	Without NAFLD	*t* or χ^2^	*p*
*n*	202	161	-	-
Sex (% men)	40.0	49.10	2.92 (χ^2^)	0.09
Age (years)	59.38 ± 11.43	60.55 ± 11.52	0.97 (*t*)	0.33
Duration of diabetes (years)	8.95 ± 6.78	9.81 ± 7.29	1.17 (*t*)	0.24
BMI (kg/m^2^)	26.76 ± 6.18	24.46 ± 14.88	8539.50 (Mann-Whitney U)	<0.001
Waist circumference (cm)	95.61 ± 10.20	87.44 ± 9.80	−7.70 (*t*)	<0.001
High blood pressure (%)	39.6	49.1	2.66 (χ^2^)	0.45
UAER (μg/min)	156.84 ± 526.30	231.18 ± 791.98	16071.00 (Mann-Whitney U)	0.85
FBG (mmol/L)	8.03 ± 2.85	7.99 ± 2.89	−0.13 (*t*)	0.89
HbA1c (%)	8.72 ± 1.89	9.12 ± 2.38	1.59 (*t*)	0.11
TC (mmol/L)	4.99 ± 1.08	4.97 ± 1.18	−0.25 (*t*)	0.8
TG(mmol/L)	2.06 ± 1.15	1.71 ± 1.30	11848.00 (Mann-Whitney U)	<0.001
HDL (mmol/L)	1.11 ± 0.30	1.30 ± 0.55	3.91 (*t*)	<0.001
LDL (mmol/L)	3.18 ± 0.93	3.12 ± 0.90	−0.71 (*t*)	0.48
AST (U/L)	24.59 ± 12.35	20.78 ± 6.61	−3.76 (*t*)	<0.001
ALT (U/L)	22.95 ± 15.52	16.44 ± 6.91	11131.50 (Mann-Whitney U)	<0.001
ABI (right)	1.03 ± 0.16	1.01 ± 0.18	6017.50 (Mann-Whitney U)	0.6
ABI (left)	1.06 ± 0.17	1.02 ± 0.20	5874.00 (Mann-Whitney U)	0.41

Values are means ± SD; differences were assessed by the unpaired *t* test (for normally distributed variables); The Mann-Whitney test (for non-normally distributed variables); and the χ^2^ test (for categorical variables).

**Table 2:** Incidence of diabetic nephropathy in participants.

	With NAFLD (*n* = 202)	Without NAFLD (*n* = 161)	χ^2^	*p*
Diabetic nephropathy	75 (37.1%)	62 (38.5%)	0.073	0.787
Microalbuminuria	51 (25.2%)	41 (25.5%)	0.502	0.778
Macroalbuminuria	24 (11.9%)	23 (14.3%)	0.50	0.480

**Table 3:** Multivariate logistic regression analyses of factors associated with diabetic nephropathy among type 2 diabetic patients.

Variables	OR	95% CI	*p*
Duration of diabetes	1.065	1.014–1.120	0.012
Waist circumference	1.077	1.040–1.116	<0.001
FBG	1.136	1.023–1.262	0.017
SEX	0.612	0.326–1.150	NS
High blood pressure	1.318	0.697–2.491	NS
TG	1.226	0.976–1.541	NS
TC	1.143	0.863–1.514	NS
ABI (left)	0.313	0.015–6.570	NS
ABI (right)	0.373	0.016–8.951	NS

Nagelkerke *R*^2^ = 0.286.
